# Asterosaponins: Structures, Taxonomic Distribution, Biogenesis and Biological Activities

**DOI:** 10.3390/md18120584

**Published:** 2020-11-24

**Authors:** Valentin A. Stonik, Alla A. Kicha, Timofey V. Malyarenko, Natalia V. Ivanchina

**Affiliations:** G.B. Elyakov Pacific Institute of Bioorganic Chemistry, Far Eastern Branch of Russian Academy of Sciences, Pr. 100-let Vladivostoku 159, 690022 Vladivostok, Russia; kicha@piboc.dvo.ru (A.A.K.); malyarenko-tv@mail.ru (T.V.M.); ivanchina@piboc.dvo.ru (N.V.I.)

**Keywords:** asterosaponins, structures, taxonomic distribution of producers, distribution in deep and shallow waters species, biological activities, biological functions, biosynthesis

## Abstract

Asterosaponins are a class of steroid oligoglycosides isolated from starfish with characteristic structures and diverse biological activities. In this review, we have attempted to combine the most important data concerning asterosaponins and give a list of these secondary metabolites with their structural peculiarities. The purpose of this review is to provide a brief but as complete as possible principal information about their chemical structures, taxonomic distribution in the marine environment, distribution in different geographical areas and depths, some properties, biological activities, and functions. Some other rare steroid metabolites from starfish, closely related in structures and probably biogenesis to asterosaponins, are also discussed.

## 1. Introduction

Asterosaponins are one of the most famous classes of marine polar steroids, discovered by Japanese chemists Y. Hashimoto and T. Yasumoto in 1960 [1]. Like some saponins of terrestrial origin, these compounds have a glycoside nature and represent a class of steroid oligoglycosides, giving foaming aqueous solutions. Asterosaponins are characteristic of one of the living classes of the phylum Echinodermata (echinoderms), namely Asteroidea (starfish or sea stars). Following Japanese scientists, a group led by an outstanding chemist Prof. L. Minale from Naples (Italy) has made a great contribution to the studies of asterosaponins [2,3,4]. Later, several teams of Russian and Chinese researchers, as well as scientists from some other countries, joined the search for and structural analysis of these secondary metabolites. Asterosaponins exhibit the characteristic hemolytic activity of saponins but differ from all other groups of saponins in their chemical structures. They demonstrate also other activities of interest in medicine.

Earlier, reviews [2,3,4,5,6,7,8,9,10,11,12] were published on marine polar steroids, including asterosaponins as one of their groups, but these articles did not contain complete information about the main features and properties of asterosaponins, accumulated to the present time, and often concerned only with specific time periods and only some aspects of their investigation.

The purpose of this review is to give a list of all these natural products known to date with their structures, taxonomic positions of producers, and places of collections. We intended to discuss the regularities in their distribution in the World Ocean, some chemical and biological properties, including biological activities, and other aspects of the investigation. We would also like to indicate new and/or such areas of the studies that should be strengthened.

## 2. Producers of Asterosaponins

Starfish are, as a rule, brightly colored marine invertebrates with a central disc and five or more arms often covered by spines. According to fossil records, starfish appeared not less than 450 million years ago. Taxonomically, the class Asteroidea (about 1900 species, inhabiting different areas from intertidal zone down to 6000 m abyssal settings in tropic, temperate, and polar waters) is difficult for analysis and many times revised. There are discrepancies and contradictions between systems based only on morphological characters and on the use of molecular genetic data. Nevertheless, classification with separation of three superorders Forcipulatacea (orders Forcipulatida and Brisingida), Spinulosacea (order Spinulosida), and Valvatacea (orders Valvatida, Paxillosida, and Notomyotida), and order Velatida are used in several taxonomic systems, including recent one [13,14]. The debate about the phylogenetic classification of the status of the infraclass Concentricycloidea (order Peripodida) continues. 

Currently, the World Asteroidea Database contains about 1890 species [13]. Among these are two orders of the superorder Forcipulatacea. The order Forcipulatida is more numerous from them with more than 280 in majority predatory species, including many well-known species from temperate regions and a few cold-water and abyssal invertebrates. Animals belonging to both this order and order Brisingida (110 species) have so-called pedicellariae, particular wrench- or claw-shaped organs capable of responding to the environment, which probably keeps clear the body surface of starfish. Representatives of Brisingida live almost exclusively in deep-sea habitats, although a few species were found in cold shallow Antarctic waters. The corresponding starfish use the suspension-feeding and tend to have softer bodies when compared with Forcipulatida. 

Three orders Valvatida, Paxillosida, and Notomyotida comprise another superorder, Valvatacea. The order Paxillosida (about 440 species) is considered by taxonomists as a primitive group. The order Valvatida includes a maximal number of species, about 760. Asterosaponins from the starfish belonging to orders Valvatida and Forcipulatida were first studied in comparison with those from other orders. The order Spinulosida (more than 130 species) contains starfish without pedicellariae and with short spines on the aboral (upper) surface. The order Velatida (about 140 species) is mostly deep-sea and cold-water starfish often of global distribution. The infraclass Concentricycloidea with 3 known species is a very small and rare group.

## 3. General Formula of Asterosaponins, Their Constituents, Some Chemical Properties

**Figure 1:**
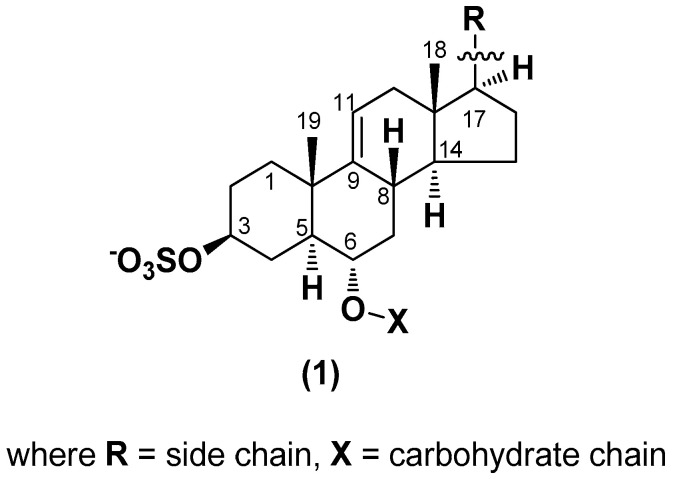
General formula of asterosaponins.

**Figure 2:**
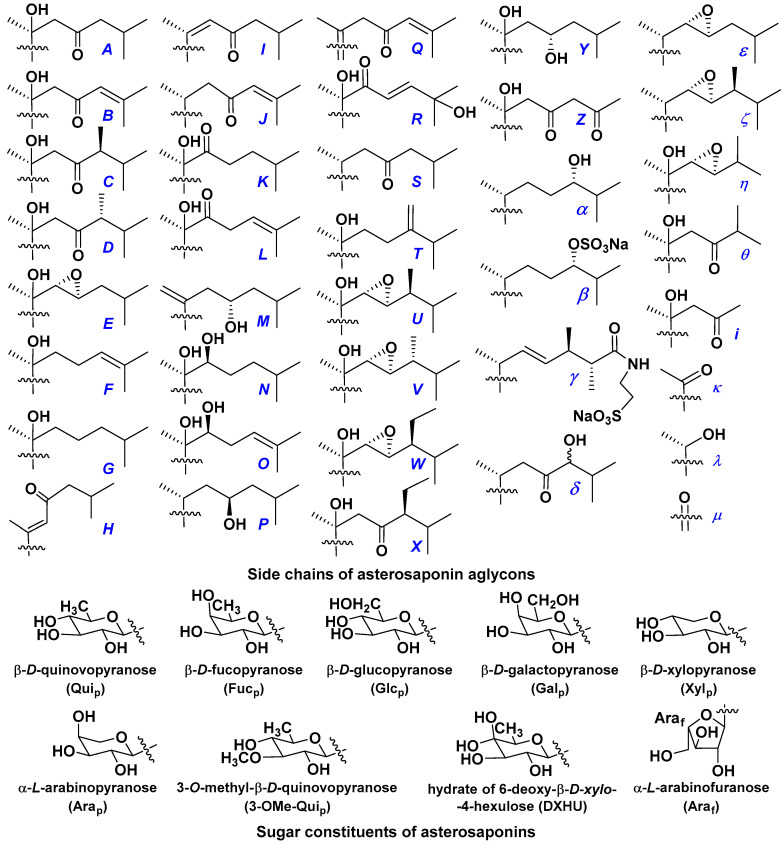
Side chains of aglycons and sugar units asterosaponins.

Chemically, classical asterosaponins are oligoglycosides with aglycons, which are Δ^9(11)^-sterol derivatives oxidized in side chains and at C-6 with sulfate group at 3β-position. Carbohydrate chains, consisting of 3–6 monosaccharides, are attached to C-6 (Figure 1). The majority of them have 5 or 6 monosaccharide residues. All these sugars are of *D*-series (beside arabinose) and attached to each other and aglycon by β-glycosidic bonds. Arabinose forms α-glycosidic bond with a neighboring sugar unit. The general formula of asterosaponins (**1**) is given below.

d-Fucopyranose (d-Fuc_p_), *D*-quinovopyranose (d-Qui_p_), d-xylopyranose (d-Xyl_p_), d-glucopyranose (d-Glc_p_), d-galactopyranose (d-Gal_p_), l-arabinopyranose (l-Ara_p_), l-arabinofuranose (l-Ara_f_), methylated d-quinovopyranose (3-OMe-d-Qui_p_), and hydrate of 6-deoxy-d-*xylo*-4-hexulose (DXHU) were found in carbohydrate chains of classical asterosaponins as sugar units. A total of 38 types of side chains in aglycon moieties were detected in them and related non-classical asterosaponins (Figure 2). 

Side chains in asterosapogenins contain 7, 8, 9, or 10 carbon atoms as in 24- or 26-*nor*-cholestane, cholane, cholestane, ergostane, and stigmastane series of sterols, respectively, and additional oxygen atoms in hydroxy, ketone, or epoxide fragments. In some cases, these structural fragments include one or rarely two double bonds.

Like other oligoglycosides, asterosaponins are hydrolyzed by acids or enzymatically with glycosidases to give either genuine aglycons or artificial genins (asterogenols) in dependence on the chemical structure of aglycons and conditions of hydrolysis along with sugar mixtures. A wide distributed aglycon, known as 3β-*O*-sulfate of thornasterol A (**2**), has the side chain, designated as A (Figure 2). It contains 20-hydroxy-23-oxo-fragment and is converted into pregnane derivative asterone (**3a**) as a result of retroaldol reaction with cleavage of C (20)–C (22) bond in the conditions of acid hydrolysis. Asterone (**3a**) and its epimer, isoasterone (**3b**), were identified in hydrolysates of many asterosaponins. Glycosides with 3β-*O*-sulfate of asterone as aglycon are considered artificial compounds in asterosaponin fractions. However, there is no complete certainty that such compounds are 100% cleavage products of native glycosides. In reality, glycosides, containing sulfated asterone, frequently but not always present in glycoside fractions of starfishes before their isolation and separation into individual asterosaponins and may be detected by mass spectrometry. Moreover, some species contain glycosides with biochemically transformed asterone aglycons having a hydroxy group at C-20 of pregnane skeleton system. Further analyses in milder conditions of extraction or using some enzymes should establish details of the origin of pregnane series asterosaponins in some or other starfish [15]. It seems that some species, which do not contain asterosaponins with side chain ***A*** (Figure 2) also contain asterosaponins with pregnane aglycons, suggesting that in these starfish glycosides containing asterone could be genuine natural products, probably formed from 20,22-dioxidized precursors in the same manner as pregnane steroid hormones are biosynthesized in higher animals. 

**Figure 3:**
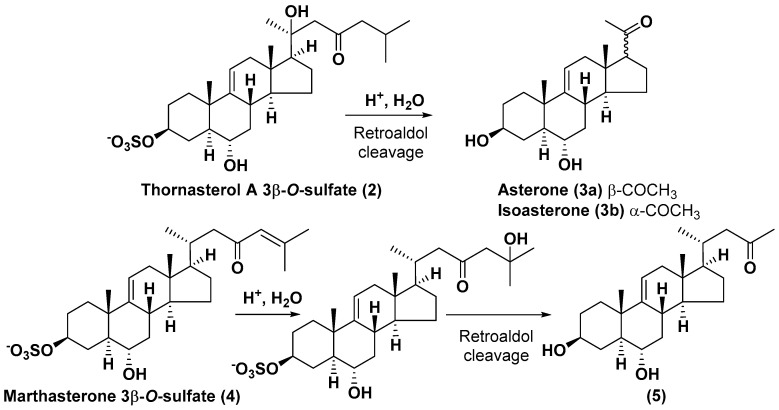
Chemical transformation of native aglycons from some asterosaponin in conditions of acid hydrolysis.

Another retroaldol type cleavage is observed, when the distributed aglycon, known as sulfate of marthasterone (**4**) with side chain ***J,*** present in asterosaponins. In such cases, cholane asterogenol (**5**) are formed at hydrolysis (Figure 3) [15]. We will discuss, along with classical asterosaponins, the structures and properties of numerous glycosides isolated from different starfish with presumably artificial genins since their structures and biological activity also may be of interest.

## 4. Structures and Taxonomic Distribution of Classical Asterosaponins

Structures of classical oligoglycoside asterosaponins, sulfated at C-3 of aglycon, and their taxonomic distributions were analyzed according to the affiliation of their producers in main orders and families of the class Asteroidea. Some information about collections of the corresponding animals is also given in the corresponding Tables. 

Diverse in respect of their chemical structures, asterosaponins were isolated from the starfish belonging to the order Valvatida with a maximal number of species in the class Asteroidea. Table 1 shows the list of these natural products, their structures, species from which they were isolated for the first time, and localities of collections of the corresponding producers. 

**Figure 4:**
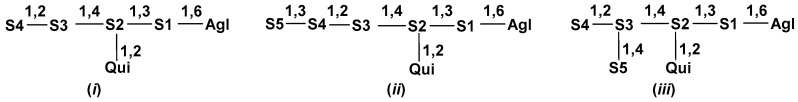
Main types of architecture of carbohydrate chains of asterosaponins.

Carbohydrate moieties show many structural features common to all compounds in this class (Figure 4). The first sugar unit (S1), attached to C-6 hydroxy group in aglycon, is quinovose in most cases. More rare carbohydrate moieties with glucose as the first monosaccharide unit were also found in some species. Next monosaccharide unit (S2) (usually quinovose or xylose) is linked to this sugar by 1,3 bond and always bears quinovose residue, attached by β-1,2-bond in this branching point. The third sugar in the main chain is attached to S2 by 1,4-bond. Terminal sugar units are connected with S3 by 1,2-bond. In a carbohydrate chain consisting of six sugars with five sugars in the main chain, a terminal sugar unit is linked with S4 by 1,3-bond (type *ii*). In hexasaccharide carbohydrate chains with two branchings, the second terminal sugar unit is linked with S3 by 1,4-bond (type *iii*). The main types of architecture of carbohydrate chains of asterosaponins from Valvatida and other starfish orders are given below.

It is of interest from chemotaxonomic point of view that rare carbohydrate chains with two branching points (type *iii*) were so far found in Valvatida only in representatives of the family Asterinidae. In contrast with the majority of asterosaponins, some oligoglycosides from starfish, belonging to the families Oreasteridae and Ophidiasteridae, contain glucose instead of quinovose as S1 unit in their carbohydrate moieties. Moreover, minor asterosaponins having only three sugars (**44**–**46**) with glucose as the first sugar were found in Oreasteridae. The loss of hydrophilicity in them is compensated by the greater polarity of aglycons. 

In their skeleton systems, the aglycon moiety of these glycosides varies from C_26_ to C_29_ compounds, although C_27_ aglycons predominate in the majority of cases, suggesting the participation of C_27_ sterols or sterol sulfates in their biosynthesis. Biotransformations, leading to these aglycons, were not studied and probably occur under the action of desaturases and oxidoreductases similar to cytochromes P_450_ in higher animals. Double bonds in 22(23)- and 24(25)-positions were indicated along with rare 20(22)- and 23(24)-double bonds. Sometimes, precursors with 22(23)-double bond give the corresponding epoxides. Characteristic patterns of oxidation at C-6, C-20, C-22, and/or C-23 were indicated in aglycons. The scientific field concerning the biosynthesis of asterosaponins and related metabolites is poorly understood, although the variety of enzymes that implement the biosynthesis of asterosaponins is significant, and some of them can be used in the biotechnology of highly oxidized steroids.

**Figure 5:**
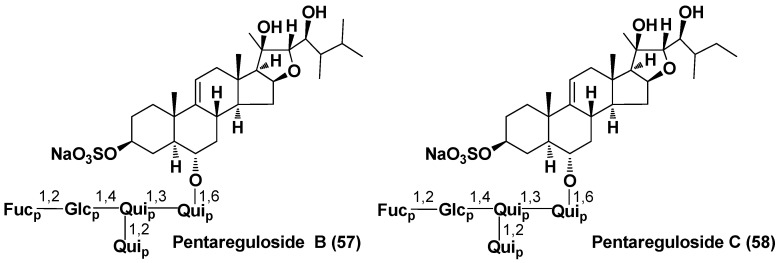
Structures of pentaregulosides B and C from the starfish *Pentaceraster regulus*.

A portion of aglycons does not contain C-23 ketone group, characteristic of many asterosaponins. Solasteroside A (**56**) with less oxidized aglycon, containing only hydroxy group at C-20 but without the ketone in the side chain, was found in *Solaster borealis* (family Solasteridae). In addition, archasteroside B (**15**) from *Archaster typicus* (family Archasteridae) and pentareguloside A (**50**) from *Pentaceraster regulus* (family Oreasteridae) have one more oxygen atom in the steroid nucleus (16β-hydroxy group). Moreover, two unprecedented oligoglycosides (**57**,**58**) from this starfish species belong to the furostane type, with **58** having a 27-*nor*-ergostane skeleton system of aglycon (Figure 5) [40].

Thus, to the best of our knowledge, 53 classical asterosaponins (together with compounds **57** and **58**) were so far isolated from starfishes belonging to the order Valvatida.

Almost all species producers of asterosaponins were found in shallow water starfish, inhabiting moderate, subtropical, and tropical areas of the Northern Hemisphere. At that, representatives of many deep-sea families either were not studied or asterosaponins were not found in them. 

The results of the studies on asterosaponins from representatives of the order Forcipulatida are given in Table 2.

The order Forcipulatida unites the most common species of the class Asteroidea. Many studied starfish were collected in the Mediterranean and other European waters, in the Far Eastern Pacific waters, and even in cold water areas adjacent to Antarctica. Thus, samples of starfish belonging to this order from both Northern and Southern Hemispheres were studied. The majority of the studied species were collected from shallow waters, although several species inhabiting depths 50–100 m were also examined and, although did not demonstrate the principal difference in structures of their asterosaponins in comparison with those of Valvatida, they often contained new variants of aglycons. A few species collected from the depths of 20–100 m contained asterosaponins with unsaturated side chains in aglycon moiety.

In the majority of cases, carbohydrate chains belong mainly to (*i*) and (*ii*) types (see Figure 4). A peculiarity of carbohydrate moieties in some asterosaponins from the corresponding starfishes consists in the presence of 6-deoxy-*xylo*-hex-4-ulose (DXHU) hydrate sugar unit, derived from quinovose. This sugar contains a ketone group in hydrate form and always occupies the first position in the carbohydrate chain. Atypical tetrasaccharide chains were found in some species of families Stichasteridae and Zoroasteridae, as well as one tetraoside and two triosides were isolated from the starfish *Asterias forbesi* (family Asteriidae).

However, only further analysis should clarify in detail the influence of deep-water environment on structures of asterosaponins. A total of 64 asterosaponins were found in the studied starfish of this order (Table 2).

The results of the studies on asterosaponins from representatives of the order Paxillosida are given in Table 3.

Starfishes belonging to the order Paxillosida often contain the characteristic of many starfish species aglycon with 20-hydroxy-23-oxo-side chain. Some of them, representatives of the families Astropectinidae and Goniopectinidae, have unusual carbohydrate moieties with rare arabinofuranose and 3-*O*-methyl-quinovopyranose sugar units and oxidized at C-20 and C-22 aglycons. Totally, 13 asterosaponins were found in starfish belonging to this order (Table 3).

The results of the studies on asterosaponins from species of the orders Spinulosida and Brisingida are given in Table 4.

The studied starfish of the order Spinulosida do not show significant differences in structures of their asterosaponins when compared with representatives of other orders. However, it does not concern two asterosaponins, collected from a significant depth (about 600 m) using a submersible. The corresponding oligoglycosides (**142**,**143**) contain very rare, oxidized only at C-20, aglycons and carbohydrate chains with two branchings. Thus, it was confirmed that deep-sea starfish may be producers of new variants of asterosaponins. It is of particular interest because only polyhydroxysteroids containing sulfate groups, but not asterosaponins, were so far found in many starfish from similar depths. To the best of our knowledge, along with 8 asterosaponins, found in Spinulosida and Brisingida, a total of 138 classical asterosaponins were isolated from starfish.

Thus, this class of secondary metabolites is characterized in Asteroidea by uniform constituents with strictly organized, numerous oligoglycoside carbohydrate moieties and diverse side chains in aglycons with a predominance of those containing both 20-hydroxy and 23-ketone functionalities. However, deep-sea species with their asterosaponins with uncommon steroid aglycons are of significant interest from point of view of the search for new variants of asterosaponins.

## 5. Structures and Distribution of Non-Classical Asterosaponins

**Figure 6:**
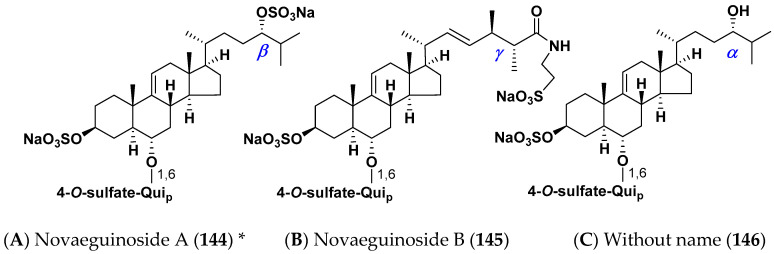
Structures of non-classical asterosaponins with one monosaccharide residue in carbohydrate chains from the starfish *Culcita novaeguineae*. (**A**) Novaeguinoside A (**144**) * [37]; (**B**) Novaeguinoside B (**145**) [37]; (**C**) Without name (**146**) [38]. * Two different glycosides are given in the literature under this name.

Several mono- and biosides, closely related to classical asterosaponins (we call them non-classical asterosaponins), were found in the starfish. One of these groups (“shortened” asterosaponins) contains only one sugar in carbohydrate moiety when compared with classical asterosaponins, but this sugar bears an additional sulfate group that partly compensates the loss in hydrophilicity. Novaeguinosides A and B and asterosaponin without a name (**144**–**146**) have 4-*O*-sulfate of quinovopyranose unit and aglycons with side chain also bearing polar substituents, as in trisaccharide asterosaponins **44**–**46** from the same starfish *Culcita novaeguinea* [37,38]. Novaeguinosides A (**144**) is the first asterosaponin with three sulfate groups (Figure 6).

Another group of closely related metabolites is monosides from the starfish (**147**–**149**), characterized by the presence of pregnane aglycons, which could not be resulted from retro-aldol cleavage from asterosaponins with 20-hydroxy and 23-oxo groups because it contains hydroxy group in the side chain of pregnane aglycons instead of carbonyl. However, co-isolated compound **150** and compound **151** have asterone (**3a**) as aglycon, but their origin from other compounds through retro-aldol reaction is questionable. Aphelasteroside C (**152**) from *Aphelasterias japonica* and latespinosides A-C (**153**–**155**) from *Astropecten latespinosus* contain aglycons with side chains earlier found in some classical asterosaponins, while latespinoside D (**156**) is the first glycoside from starfish with androstane type of aglycon (Table 5).

**Figure 7:**
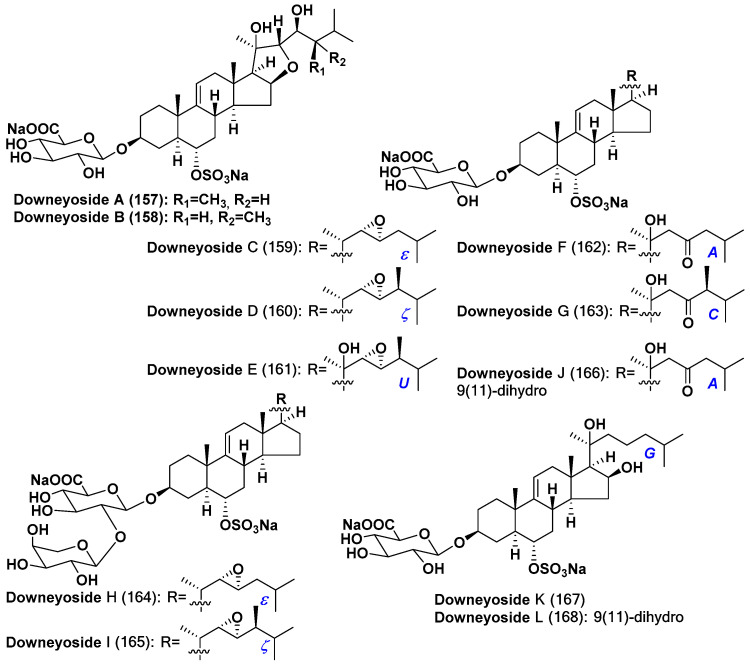
Structures of downeyosides A–L from the starfish *Henricia downeyae*.

One more group of starfish polar steroids, downeyosides A–L (**157**–**168**), also possesses the structural similarity to asterosaponins and has the same type of ∆^9(11)^ disubstituted 3β,6α-dihydroxy steroid aglycons, but in the contrast with classical asterosaponins, carbohydrate moiety in them is attached to C-3 and a sulfate group is located at C-6 (Figure 7) [84,85]. These steroid metabolites were isolated from the starfish *Henricia downeyae* (order Spinulosida, family Echinasteridae), collected in the northern Gulf of Mexico at the depth of 90 m. These glycoside sulfates also contain a monosaccharide with a polar substituent, namely glucuronic acid. Some of downeyosides are monosides, but downeyosides H and I (**164**,**165**) contain an additional *L*-arabinose unit. Downeyosides A (**157**) and B (**158**) contain the fifth ring in the steroid nucleus, in which C-16 and C-22 are linked through ethereal oxygen. Downeyosides K (**167**) and L (**168**) have additional oxidation at C-16 in the ring D. Downeyosides J (**166**) and L (**168**) are 9(11)-dihydro derivatives [84,85].

**Figure 8:**
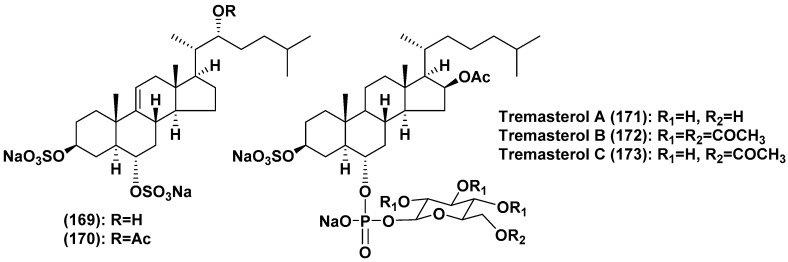
Structures of disulfates and tremasterols A-C from the starfish *Tremaster novaecaledoniae*.

Thus, more than 160 asterosaponins and closely related metabolites were found from starfish. These polar steroids were isolated from representatives of all the studied orders of the class Asteroidea, collected in the majority of cases in shallow waters of the World Ocean. Deep-sea species were rarely analyzed [86,87] and classical, but unusual asterosaponins (**142**,**143**) were isolated from *Novodinia antillensis* [78], collected from a depth of 587 m, while asterosaponin-like compounds (**157**–**168**) were found in *Henricia downeyae* from a depth of 90 m [84,85]. On the other hand, polyhydroxylated steroid sulfates seem to be more characteristic metabolites of deep-sea starfish. There are several unique polar steroids among them such as similar to asterogenol disulfates **169** and **170**, containing sulfate groups at the both 3β- and 6α-positions, and tremasterols A-C (**171**–**173**), possessing carbohydrate residues attached to C-6 through phosphate bridge, from a «living fossil» starfish *Tremaster novaecaledoniae* (order Valvatida, family Asterinidae) (Figure 8) [88,89]. Further studies of polar steroids from deep-sea starfish are one of the promising directions of the search for new asterosaponins and other bioactive marine steroids.

## 6. Biogenesis and Biological Functions of Asterosaponins

The processes of the biosynthesis of asterosaponins are still extremely poorly understood, despite the fact that the first attempts to clarify it were made in the seventies of the last century. The first question, concerning with principles of their biogenesis, is whether *de novo* biosynthesis of sterols, precursors of aglycons of these compounds in starfish, is possible or these aglycons are formed in starfish from sterols obtained by these invertebrates from the diet. In 1977, Mackie et al. carried out labeling experiments with [2-^14^C]-mevalonic acid and [4-^14^C]-cholesterol and established that the starfish *Marthasterias glacialis* can synthesize asterosaponins both *de novo* and from dietary sterols, although the incorporation was very low, particularly from cholesterol [90]. Later, similar results were obtained by Australian biochemists at the studies on asterosaponins from the starfish *Patiriella calcar* [91]. They have also shown that this animal can synthesize steroidal saponins from both [2-^14^C]-mevalonic acid and [4-^14^C]-cholesterol, therefore, through biosynthesis *de novo* and transformation of dietary sterols into these metabolites. 

Another important discovery was the finding that cholesterol is converted in the starfish *Asterias rubens* into cholest-7-enol (a principal free sterol in starfish and holothurians) through cholestanol. Moreover, conversion of cholest-7-enol into the corresponding epoxide was also indicated [92]. This process could lead to some polar steroids of starfish, particularly to mono- and biosides of polyhydroxysteroids. It was also found that *Asterias rubens* is able to introduce a double bond at C-22 in both cholesterol and cholest-7-enol [93]. Derivatives, containing 22(23)-double bond and the corresponding epoxides [94], were many times found among asterosaponins (see Table 1, Table 2, Table 3 and Table 4). 

It was shown that sterols in Asteroidea and Holothuroidea, whose representative contain oligoglycosides, and on less level in other classes of Echinodermata, are converted into steryl sulfates. C_27_-components predominate among sulfated metabolites of starfish and in asterosaponins, even when C_28_- and C_29_-compounds are present as the major free sterols of a starfish [94,95]. Such sulfated metabolites are very likely suggested as biosynthetic precursors of asterosaponins, although experimental confirmations are absent at the present time.

Cytochrome P450 monooxygenases are obligate biochemical systems providing steroid metabolites with hydroxyl groups in steroid nucleus and side chains. Participation of these enzymes in the biosynthesis of polar steroids in starfish seems to be very probable. It is of interest that the results of a limited number of studies on echinoderms have provided evidence for the presence of a cytochrome P450 monooxygenase system in starfish [96].

Thus, the scientific field concerning the biosynthesis of asterosaponins is waiting for main discoveries in the next future. First of all, it is necessary to find out: (*i*) whether free sterols or their 3-*O*-sulfates are the main biosynthetic precursors of asterosaponins, (*ii*) how hydroxy group is introduced into C-6 of their molecules, (*iii*) what enzymes are involved in side chain transformations, and (*iv*) in what organs and body parts this biosynthesis occurs.

Recently, Ivanchina et al. [97], using deuterium-labeled precursors in feeding experiments, have shown that the conversion of free cholesterol and its 3β-*O*-sulfate into polyhydroxylated steroids, containing 6α-hydroxy group, proceeds via cholest-4(5)-en-3-one intermediate in the starfish *Patiria (=Asterina) pectinifera*. However, it is not known whether the same sequence of transformations is realized in the biosynthesis of asterosaponins.

Biological functions attracted attention right away after the discovery of asterosaponins. Three main properties were particularly attractive: (1) participation in chemical protection of these invertebrates against predators, (2) influence on the maturation of sexual products before the breeding season, (3) their solubilizing role in relation to rich in lipids and sterols diet.

Asterosaponin-containing extracts proved to be highly ichthyotoxic and hemolytic. Toxicity against fish, mollusks, annelids, and arthropods was described [98,99,100]. The high content of saponins in such body constituents as body walls and stomach also suggest their protective role (particularly taking into attention that in many species during hunting the stomach extends beyond the body to digest the victim and itself becomes vulnerable to predators). Additionally, these compounds were reported as protectors of their producers against parasites and microbial pathogens. Moreover, these toxins are sometimes active in catching prey [101]. Recently, it was demonstrated that saponins are located not only inside the body wall of the animals but also within the mucus layer that probably protects the animal against external aggressions [102]. Thus, the most probable biological function of these metabolites in many cases is the protective one.

On the other hand, the majority of starfish are themselves predators and their victims (mollusks and other small animals) are able to perceive the increasing concentration of asterosaponins as they approach and respond to this with characteristic avoidance reactions quickly moving away from a predator. Mackie et al. have studied this «escape reaction» caused by some asterosaponins particularly against dietary mollusks [103].

Another hypothesis suggests that asterosaponins act as spawning inhibitors in the ovary of starfish. Synchronization of egg maturation has a great significance for the successful reproduction of starfish species. It was shown that levels of asterosaponins demonstrate dynamics connected with the breeding season in the starfish *Asterias amurensis*. The content of these metabolites is higher in summer than in winter [104]. Moreover, these toxins inhibit the maturation of oocytes [105]. That is why it was suggested that asterosaponins could synchronize the maturation of oocytes before fertilization. However, in contrast with *A. amurensis*, the breeding season of which started in February-March, when the level of asterosaponins is lower, in some other starfish such as *A. vulgaris*, *A. rubens*, and *Marthasterias glacialis,* high levels of asterosaponins were detected during the breeding season. Therefore, the primary functions of asterosaponins cannot be spawning inhibition in all starfish species [106].

Nevertheless, the important role of asterosaponins in the reproduction of starfish was also confirmed by other data. In the middle of the last century, American scientist Dan carried out a research program on the fertilization of marine invertebrates at Misaki Marine Biological Station and discovered the biological significance of the changes, which introduced the term “acrosome reaction.” The acrosome reaction «is the mechanism for spermatozoa to ensure a spatio-temporally matched exposure of devices essential for penetration through the egg coat and for subsequent fusion with egg plasma membrane [107]. Spawned eggs of starfish have two layers of acellular coats. A transparent and gelatinous outer layer (jelly coat) consists of highly sulfated glycoproteins named as acrosome reaction-inducing substance (ARIS), as well as of a group of sulfated steroidal glycosides (asterosaponins) named as Co-ARIS, and sperm-activating peptides (SAP). Co-ARIS glycosides proved to be asterosaponins. These compounds, containing the hydrated form of 6-deoxy-D-*xylo*-4-hexulose in carbohydrate moiety, act as co-factors for ARIS to induce along with SAP acrosome reaction and, therefore, play an important role in fertilization.

Other polar steroids such as monoglycosides of polyhydroxysteroids showed a high and fairly constant composition in digestive tissues of the starfish *P. pectinifera* in spite of small seasonal variations in the relative concentrations of individual compounds [108,109]. Glycosides of polyhydroxysteroids prevailed in pyloric caeca of this starfish as well as of *Lethasterias fusca*, suggesting that this group of steroid metabolites, rather than asterosaponins, may participate in the assimilation of rich in lipids diet of these animals [110]. 

## 7. About Biological Activities of Asterosaponins

The aim of this short part of our review is an attempt to focus on more promising types of asterosaponin activities and on such directions of further investigation, which, in our opinion, could be prospective to find new useful properties and applications. Recently, several reviews [12,111,112] were published about the biological activities of polar steroids from starfish, including asterosaponins.

### 7.1. Cytotoxic and Cytostatic Actions

The majority of the studies on different biological activities of asterosaponins were carried out, first of all, in relation to their cytotoxic properties against cancer cells. In many cases, only slight or moderated effects were found with IC_50_ values ranged from several dozen µM down to 5 µM. However, there were several cases when more potent cytotoxicity against different lines of cancer cell lines was indicated. High activity was connected either with structural peculiarities in side chains of aglycons or structures of carbohydrate moieties of asterosaponins. For example, leptasterioside A (**103**) [62], having a 20-hydroxy group in 24-methylene-containing side chain in its aglycon, demonstrated IC_50_ value of 2 µM against T-47D tumor cells. Antarcticosides A–C (**137**–**139**), containing hexasaccharide carbohydrate chains with two branches (type *ii*, Figure 4) from a starfish of the family Echinasteridae collected in the Antarctic Sea, was cytotoxic against human bronchopulmunary non-small-cell lung carcinoma at IC_50_ less than 3.3 mg/mL [77]. A list of asterosaponins that showed promising activity against tumor cells is given in Reference [111]. Some interesting examples of the anticancer action of asterosaponins were also reported in the review of Katanaev et al. [112].

Molecular mechanisms of cytotoxic effects were studied in several cases. Novaeguinosides A–D (**144**,**145**,**44**,**45**) showed the promotion of tubulin polymerization in tumor cells and cytotoxicity against human leukemia K-562 and human hepatoma BEL-7402 cells [37]. Archasterosides A and B (**14**,**15**) from the starfish *Archaster typicus* induced P53- and AP-1 (activating protein-1)-independent apoptosis of tumor cells [21]. Astrosterioside D (**126**) showed induction of apoptosis via the inactivation of PI3K/AKT and ERK 1/2 MAPK signal pathways and down-regulation of the protein C-myc [70,111]. Cytostatic action was established against eggs and sperm of sea urchins (as a rule, embryos of the sea urchin *Strongylocentrotus intermedius* were used as a model system). Asterosaponins, like holostane glycosides from sea cucumbers, exhibit the blocking of the embryo’s development and inhibit fertilization by sperm of this sea urchin [12].

In many cases, asterosaponins not only suppress cell proliferation but also inhibit colony formation in tumor cells acting at non-toxic or low toxic concentrations [12]. For example, lethasterioside A (**110**) from *Lethasterias fusca* strongly suppresses colony formation of T-47D, RPMI-7951, and HCT-116 tumor cells in soft agar clonogenic assay at a concentration of 20 μM [64]. Hippasterioside D (**30**) from the starfish *Hippasteria kurilensis* shows inhibition of colony formation of the HT-29 tumor cells [29]. This compound at the dose of 60 μg/mL suppressed the size of colonies, although colony numbers were only moderately suppressed [29,112]. Acanthaglycoside A (**7**) and luidiaglycoside B (**130**) effectively inhibited colony formation of tumor cells at non-cytotoxic concentrations and prevented migration HT-29 and MDA-MB-231 cells [20]. These results suggested that asterosaponins might be prospective in in vivo studies on tumors with the application of combinations of known antitumor drugs and asterosaponins as potential inhibitors of metastasis.

### 7.2. Antimicrobial Action

Some asterosaponins demonstrate antifungal action against the plant pathogenic fungus *Cladosporium cucumerinum*. For example, anasteroside A (**59**) and versicoside A (**73**) showed perceptible inhibition zones on this fungus at a concentration of 10 µg of a tested compound in a spot. Desulfation of versicoside A by solvolysis in dioxane/pyridine (1:1) gave a totally inactive saponin [43]. Antifungal activities of some other asterosaponins were also reported [113]. Ethanolic extracts of the starfish *Henricia downeyae*, which contain non-classical asterosaponins with glucuronic acid in their carbohydrate moieties, such as **157**–**168** [84,85], caused growth inhibition of bacteria and fungi, and potent antifouling activity. However, it is unknown whether purified glycosides can exhibit this biological action.

### 7.3. Anti-Inflammatory Action

There are numerous reports concerning with capability of asterosaponins to induce the production of different pro-inflammatory or anti-inflammatory cytokines. Macrophages RAW 264.7 were frequently used in the search for asterosaponins with anti-inflammatory properties [12]. The studied asterosaponins either increased the production of reactive oxygen species (ROS) by these macrophages, stimulating their activity, or decreased the level of ROS after stimulation of the macrophages by lipopolysaccharide from *E. coli*, thus decreasing hyperstimulation. Other model cells were also used. For example, astrosteriosides A, D (**123**,**126**), anti-inflammatory asterosaponins from the starfish *Astropecten monacanthus,* the edible species collected in Vietnamese waters, and marthasteroside B (**114**) demonstrated potent anti-inflammatory activity comparable with positive control with treatment by SB 203,580 at measuring the production of pro-inflammatory cytokines interleukin-12 (Il-12 p40), interleukin-6 (IL-6), and tumor necrosis factor-α (TNFα) in lipopolysaccharide-stimulated bone marrow-derived dendritic cells [70].

## 8. Conclusions

To the best of our knowledge, as a result of sixty years of the studies, 128 classical asterosaponins, being oligoglycosides with 3β-*O*-sulfated 9(11)-unsaturated steroid aglycons of cholestane, 24- or 26-*nor*-cholestane, cholane, ergostane, or stigmastane series, and 10 related, possibly artificial compounds with pregnane aglycons, were isolated from representatives of five orders of the class Asteroidea. These species were collected mainly in shallow waters, but several deep-sea starfish were also studied. In addition, 28 closely related compounds called as non-classical asterosaponins were also isolated and differed from the majority of the saponins in shortened carbohydrate moieties or even in sulfation at C-6 and glycosylation at C-3 in 9(11)-unsaturated steroid aglycons. Asterosaponins are a compact group of metabolites with predominated 20-oxy and 23-oxo functions in the side chain and three types of architecture of carbohydrate chains usually consisting of 5 or 6 monosaccharide units. These compounds carry out important biological functions connected with protection against predators and parasites, participation in reproductive processes, and probably facilitating ingestion. Biosynthesis of these marine natural products is poorly studied, and the main enzymes catalyzing the corresponding processes and consequences of biosynthetic transformations are unknown. There are many taxa of Asteroidea, particularly among deep-sea inhabitants, which were not so far investigated. It promises the discovery of new structural variants of asterosaponins, because first attempts to study such species seem to show more saturated side chains in aglycons. Moreover, other polar steroids from deep-sea starfish usually contain additional sulfates and such unusual steroids group as phosphate. Although Chinese folk medicine postulates tonic, anticancer, and anti-inflammatory properties of bioactive metabolites from starfish, the corresponding bioactivities of asterosaponins remain to be unstudied on in vivo level.

## Figures and Tables

**Table 1:** Structures and taxonomic distribution of asterosaponins from starfish of the order Valvatida.

№	Name	Side Chain (R)	Carbohydrate Chain (X)	Collection	References
Family Acanthasteridae *Acanthaster planci*					
**6**	Thornasteroside A (=Ophidianoside E)	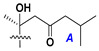	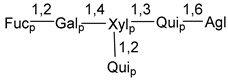	Okinawa, Japan, shallow waters	[16]
**7**	Acanthaglycoside A	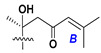	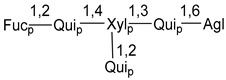	Okinawa, Japan, shallow waters	[17]
**8**	Acanthaglycoside B	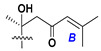	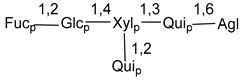	Okinawa, Japan, shallow waters	[18]
**9**	Acanthaglycoside C	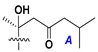	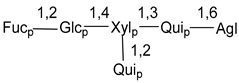	_«-----------»_	[18]
**10**	Acanthaglycoside D	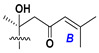	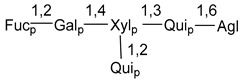	_«-----------»_	[18]
**11**	Acanthaglycoside F	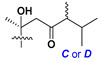	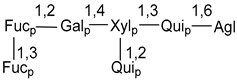	_«-----------»_	[18]
**12**	Without name (=Asterone analog of thornasteroside A)	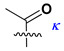	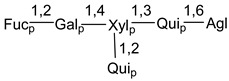	Bungo Channel, Ehime Prefecture, Japan, depth was not reported	[19]
**13**	Acanthaglycoside G	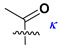	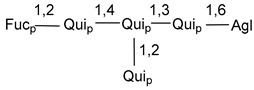	Van Phong Bay, Vietnam, depth of 5–10 m	[20]
Family Archasteridae *Archaster typicus*					
**14**	Archasteroside A	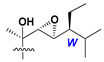	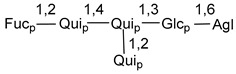	Quang Ninh, Vietnam, shallow waters	[21]
**15**	Archasteroside B (16β-hydroxy)	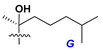	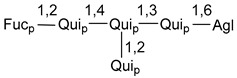	_«-----------»_	[21]
**16**	Archasteroside C	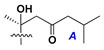	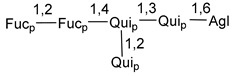	_«-----------»_	[22]
Family Asterinidae *Patiria pectinifera (Asterina pectinifera)*					
**17**	Pectinioside A	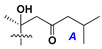	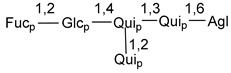	Fukuoka, Japan, shallow waters	[23]
**18**	Pectinioside B	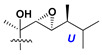	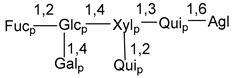	_«-----------»_	[23]
**19**	Pectinioside C	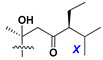	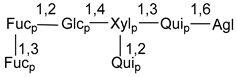	_«-----------»_	[24]
**20**	Pectinioside D (=Asterone analog of pectinioside A)	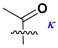	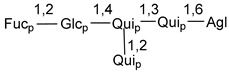	_«-----------»_	[24]
**21**	Pectinioside E	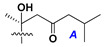	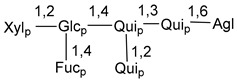	_«-----------»_	[25]
**22**	Pectinioside F	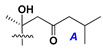	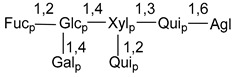	_«-----------»_	[25]
**23**	Pectinioside G	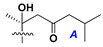	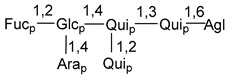	_«-----------»_	[26]
*Patiria miniata*					
**24**	Patirioside A	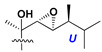	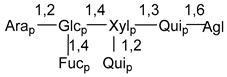	Gulf of California, shallow waters	[27]
**25**	Patirioside B	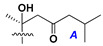	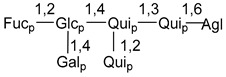	_«-----------»_	[27]
Family Asteropseidae *Asteropsis carinifera*					
**26**	Asteropsiside A	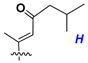	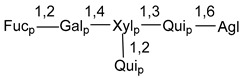	Van Fong Bay, Vietnam, shallow waters	[28]
Family Goniasteridae *Hippasteria kurilensis*					
**27**	Hippasterioside A	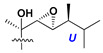	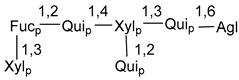	Matua Isl., Sea of Okhotsk, depth of 100 m	[29]
**28**	Hippasterioside B	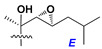	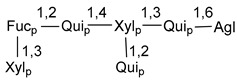	_«-----------»_	[29]
**29**	Hippasterioside C	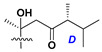	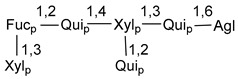	_«-----------»_	[29]
**30**	Hippasterioside D	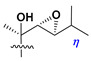	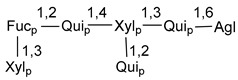	_«-----------»_	[29]
Family Ophidiasteridae *Linckia laevigata*					
**31**	Laevigatoside	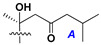	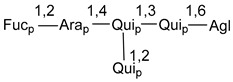	_«-----------»_	[30]
*Ophidiaster ophidianus*					
**32**	Ophidianoside B	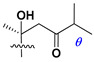	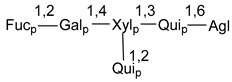	Bay of Naples, Italy, shallow waters	[31]
**33**	Ophidianoside C	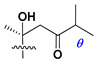	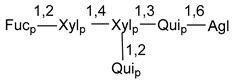	_«-----------»_	[31]
**34**	Ophidianoside F	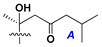	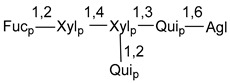	_«-----------»_	[31]
Family Oreasteridae *Halityle regularis*					
**35**	Regularioside A	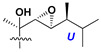	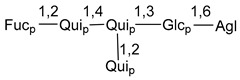	New Caledonia, shallow waters	[32]
**36**	Regularoside B	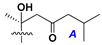	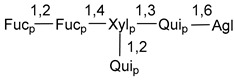	_«-----------»_	[32]
*Culcita novaeguineae*					
**37**	Novaeguinoside I	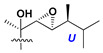	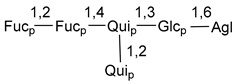	Sanya Bay, China, depth 2–15 m	[33]
**38**	Novaeguinoside II	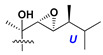	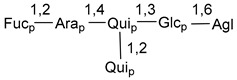	_«-----------»_	[33]
**39**	Without name (Asterosaponin 1)	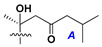	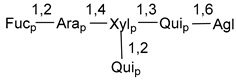	_«-----------»_	[34]
**40**	Without name	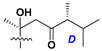	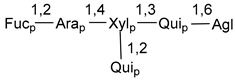	_«-----------»_	[34]
**41**	Without name	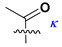	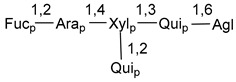	_«-----------»_	[34]
**42**	Without name	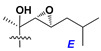	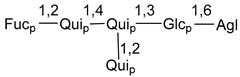	_«-----------»_	[35]
**43**	Novaeguinoside A (two different glycosides are given in the literature under this name)	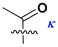	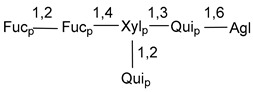	Sanya Bay, China, depth was not reported	[36]
**44**	Novaeguinoside C	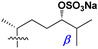	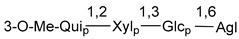	Sanya Bay, China, depth 2–20 m	[37]
**45**	Novaeguinoside D	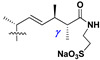	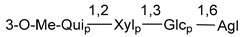	_«-----------»_	[37]
**46**	Without name	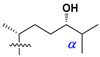	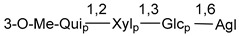	_«-----------»_	[38]
*Oreaster reticulatus*					
**47**	Reticulatoside A	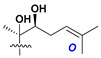	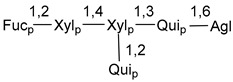	Grand Bahama Isl., Bahamos, depth was not reported	[39]
**48**	Reticulatoside B	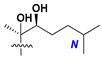	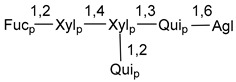	_«-----------»_	[39]
**49**	Asterone analog of ophidianoside F	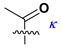	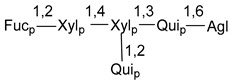	_«-----------»_	[39]
*Pentaceraster regulus*					
**50**	Pentareguloside A (16β-hydroxy)	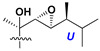	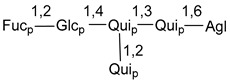	Cham Isls., Vietnamdepth of 5–20 m	[40]
**51**	Pentareguloside D	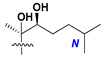	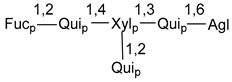	_«-----------»_	[40]
**52**	Pentareguloside E	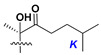	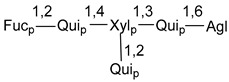	_«-----------»_	[40]
**53**	Pentareguloside F	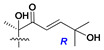	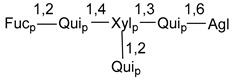	_«-----------»_	[40]
**54**	Pentareguloside G	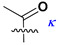	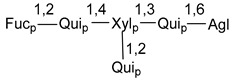	_«-----------»_	[40]
*Protoreaster nodosus*					
**55**	Protoreasteroside	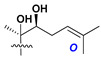	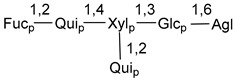	New Caledonia, shallow waters	[41]
Family Solasteridae *Solaster borealis*					
**56**	Solasteroside A	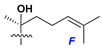	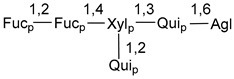	Mutsu Bay, Japan, depth was not reported	[42]

**Table 2:** Structures and taxonomic distribution of asterosaponins from starfish belonging to the order Forcipulatida.

№	Name	Side Chain (R)	Carbohydrate Chain (X)	Collection	References
Family Asteriidae *Anasterias minuta*					
**59**	Anasteroside A	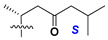	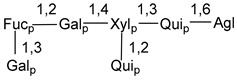	Argentine Patagonian coast, depth was not reported	[43]
**60**	Anasteroside B	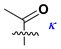	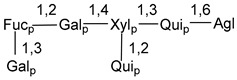	_«-----------»_	[43]
*Aphelasterias japonica*					
**61**	Aphelasteroside F	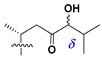	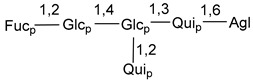	Posyet Bay, Sea of Japan, Russia, depth of 3–10 m	[44]
*Asterias amurensis*					
**62**	Glycoside B_2_ (=Forbeside B)	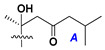	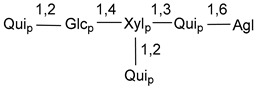	Pacific coast of Hokkaido, Japan, depth was not reported	[45]
**63**	Ovarian asterosaponin-1 (=Co-Aris I, =Forbeside C)	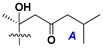	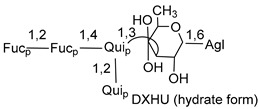	_«-----------»_	[46]
**64**	Co-ARIS II	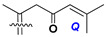	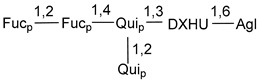	_«-----------»_	[47]
**65**	Ovarian asterosaponin-4 (=Co-Aris III)	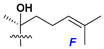	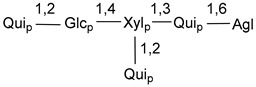	_«-----------»_	[48]
**66**	Asteroside A	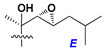	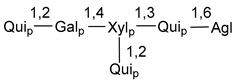	Pacific coast of Hokkaido, Japan, depth was not reported	[49]
**67**	Asteroside B	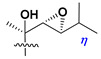	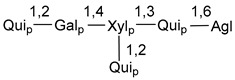	_«-----------»_	[49]
**68**	Asteroside C	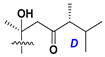	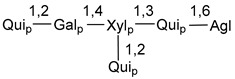	_«-----------»_	[49]
**69**	Asteroside D	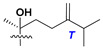	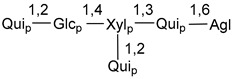	_«-----------»_	[49]
**70**	Without name	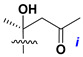	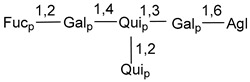	Coast of Pohang, Korea, depth was not reported	[50]
**71**	Without name	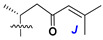	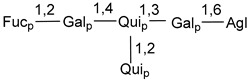	_«-----------»_	[50]
**72**	Without name	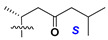	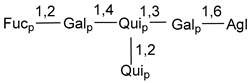	_«-----------»_	[50]
*Asterias amurensis* [cf.] *versicolor*					
**73**	Versicoside A (=Forbeside A)	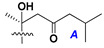	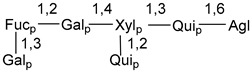	Nagasaki Prefecture, Japan, offshore of Shimabara	[51]
**74**	Versicoside B	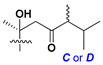	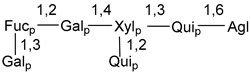	_«-----------»_	[52]
**75**	Versicoside C	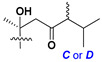	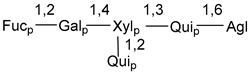	_«-----------»_	[52]
*Asterias forbesi*					
**76**	Forbeside D	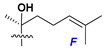	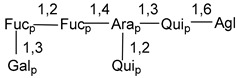	Passamaquoddy Bay, Canada	[53]
**77**	Forbeside F	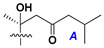	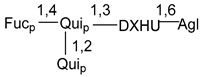	_«-----------»_	[54]
**78**	Forbeside G	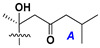	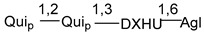	_«-----------»_	[54]
**79**	Forbeside H	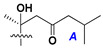	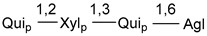	_«-----------»_	[54]
*Asterias rubens*					
**80**	Ruberoside A	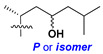	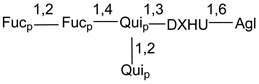	Fredericia, Denmark, Baltic Sea, depth was not reported	[55,56]
**81**	Ruberoside B	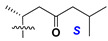	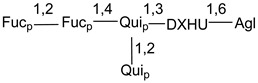	_«-----------»_	[55,56]
**82**	Ruberoside C	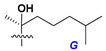	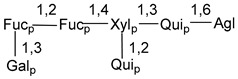	_«-----------»_	[55,56]
**83**	Ruberoside D	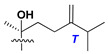	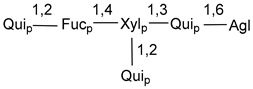	_«-----------»_	[55,56]
**84**	Ruberoside E	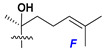	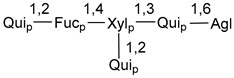	_«-----------»_	[56,57]
**85**	Ruberoside F	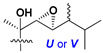	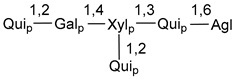	_«-----------»_	[56,57]
**86**	Ruberoside G	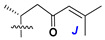	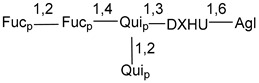	_«-----------»_	[56]
**87**	Without name	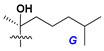	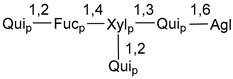	_«-----------»_	[55,56]
*Unidentified species of the family Asteriidae*					
**88**	Asteriidoside A	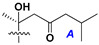	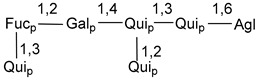	Tethys Bay, Antarctica, depth was not reported	[58]
**89**	Asteriidoside B	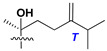	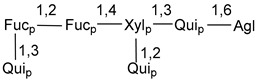	_«-----------»_	[58]
**90**	Asteriidoside C	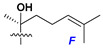	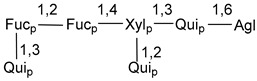	_«-----------»_	[58]
**91**	Asteriidoside D	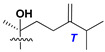	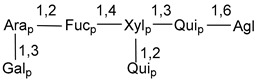	_«-----------»_	[58]
**92**	Asteriidoside E	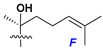	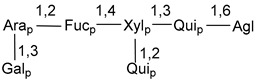	_«-----------»_	[58]
*Coscinasterias tenuispina*					
**93**	Tenuispinoside A	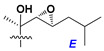	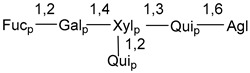	Bay of Naples, Italy, depth was not reported	[59]
**94**	Tenuispinoside B	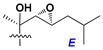	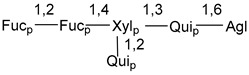	_«-----------»_	[59]
**95**	Tenuispinoside C(12α-hydroxy)	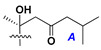	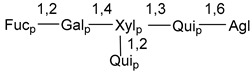	_«-----------»_	[59]
**96**	Asterone analog of regularoside B	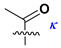	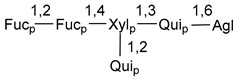	_«-----------»_	[59]
*Diplasterias brucei*					
**97**	Diplasterioside A	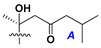	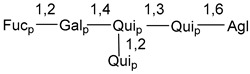	Terra Nova Bay, Antarctica, depth was not reported	[60]
**98**	Diplasterioside B	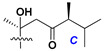	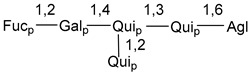	_«-----------»_	[60]
*Distolasterias nipon*					
**99**	Nipoglycoside A	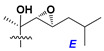	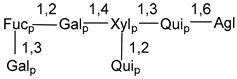	Mutsu Bay, Japan, depth was not reported	[61]
**100**	Nipoglycoside B	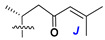	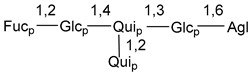	_«-----------»_	[61]
**101**	Nipoglycoside C	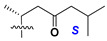	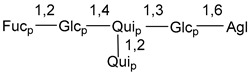	_«-----------»_	[61]
**102**	Nipoglycoside D	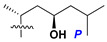	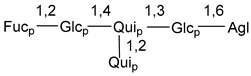	_«-----------»_	[61]
*Leptasterias ochotensis*					
**103**	Leptasterioside A	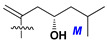	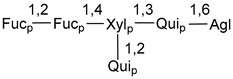	Isl. Bolshoy Shantar, Sea of Okhotsk, depth 20–40 m	[62]
**104**	Leptasterioside B	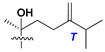	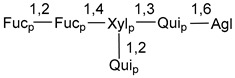	_«-----------»_	[62]
**105**	Leptasterioside C	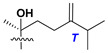	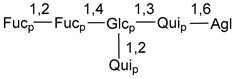	_«-----------»_	[62]
**106**	Leptasterioside D	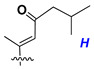	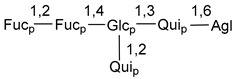	_«-----------»_	[62]
**107**	Leptasterioside E	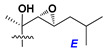	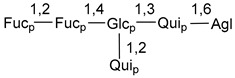	_«-----------»_	[62]
**108**	Leptasterioside F	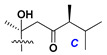	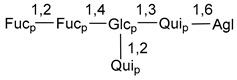	_«-----------»_	[62]
*Leptasterias hylodes reticulata*					
**109**	Hylodoside A	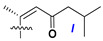	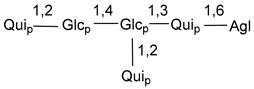	Sea of Okhotsk, depth 50–100 m	[63]
*Lethasterias fusca*					
**110**	Lethasterioside A	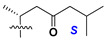	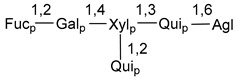	Posyet Bay, Sea of Japan, Russia, depth 5–10 m	[64]
**111**	Lethasterioside B	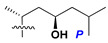	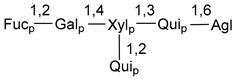	_«-----------»_	[64]
*Marthasterias glacialis*					
**112**	Marthasteroside A_1_	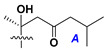	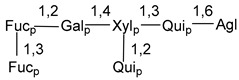	Bay of Naples, Italy, depth was not reported	[65]
**113**	Marthasteroside A_2_	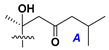	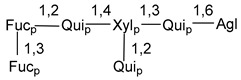	_«-----------»_	[65]
**114**	Marthasteroside B	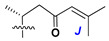	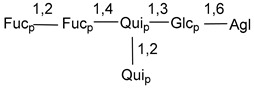	_«-----------»_	[65]
**115**	Marthasteroside C	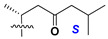	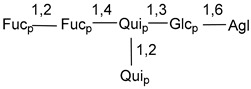	_«-----------»_	[65]
Family Heliasteridae *Labidiaster annulatus*					
**116**	Labidiasteroside A	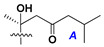	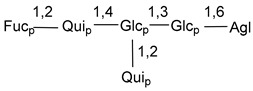	Antarctic waters	[66]
Family Stichasteridae *Cosmasterias lurida*					
**117**	Cosmasteroside A	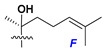	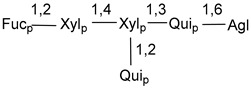	Argentine Patagonian coast, depth was not reported	[67]
**118**	Cosmasteroside B	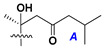	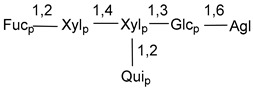	_«-----------»_	[67]
**119**	Cosmasteroside C	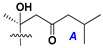	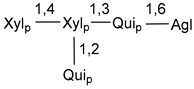	_«-----------»_	[67]
**120**	Cosmasteroside D	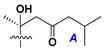	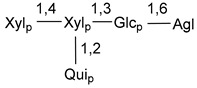	_«-----------»_	[67]
*Neosmilaster georgianus*					
**121**	Santiagoside	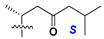	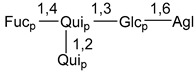	Greenwich Isl., Santiago, Chile, depth 35 m	[68]
Family Zoroasteridae *Myxoderma platyacanthum*					
**122**	Myxodermoside A	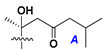	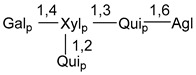	Gulf of California, depth was not reported	[69]

**Table 3:** Structures and taxonomic distribution of asterosaponins from starfish belonging to the order Paxillosida.

№	Name	Side Chain (R)	Carbohydrate Chain (X)	Collection	References
Family Astropectinidae *Astropecten monacanthus*					
**123**	Astrosterioside A	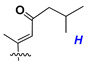	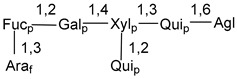	Cat Ba, Haiphong, Vietnam, depth was not reported	[70]
**124**	Astrosterioside B	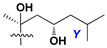	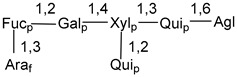	_«-----------»_	[70]
**125**	Astrosterioside C	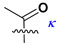	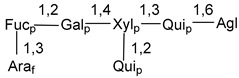	_«-----------»_	[70]
**126**	Astrosterioside D	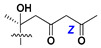	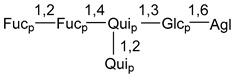	_«-----------»_	[70]
*Psilaster cassiope*					
**127**	Psilasteroside	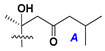	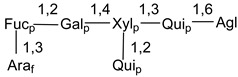	Northern Gulf of Mexico, offshore waters	[71]
Family Luidiidae *Luidia quinaria*					
**128**	Luidiaquinoside	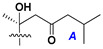	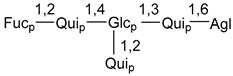	Sendai, Japan, depth was not reported	[71]
*Luidia maculata*					
**129**	Without name (Japanese group)	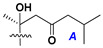	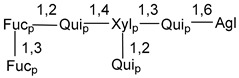	Fukuoka Prefecture, Japan, depth was not reported	[72]
**130**	Maculatoside (Italian group) (=Luidiaglycoside B)	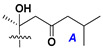	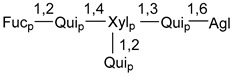	Noumea, New Caledonia, depth was not reported	[73]
**131**	Luidiaglycoside C	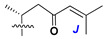	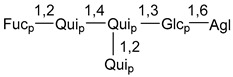	Fukuoka Prefecture, Japan, depth was not reported	[74]
**132**	Luidiaglycoside D	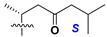	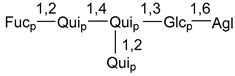	_«-----------»_	[74]
Family Goniopectinidae *Goniopecten demonstrans*					
**133**	Goniopectenoside A	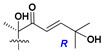	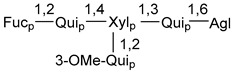	Deep waters of the Gulf of Mexico	[75]
**134**	Goniopectenoside B	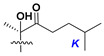	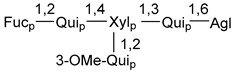	_«-----------»_	[75]
**135**	Goniopectenoside C	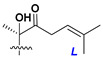	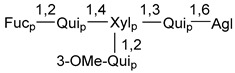	_«-----------»_	[75]

**Table 4:** Structures and taxonomic distribution of asterosaponins from starfish belonging to the orders Spinulosida and Brisingida.

№	Name	Side Chain (R)	Carbohydrate Chain (X)	Collection	References
Order Spinulosida Family Echinasteridae *Echinaster brasiliensis*					
**136**	Brasiliensoside	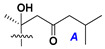	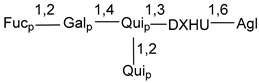	Grand Bahama Isl., depth was not reported	[76]
*Unidentified species of the family Echinasteridae*					
**137**	Antarcticoside A	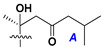	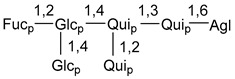	Tethys Bay, Antarctica, depth was not reported	[77]
**138**	Antarcticoside B	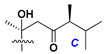	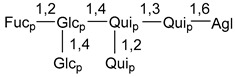		[77]
**139**	Antarcticoside C	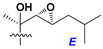	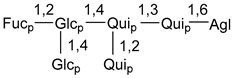	_«-----------»_	[77]
**140**	(24*S*)-Methyl- brasiliensoside	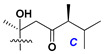	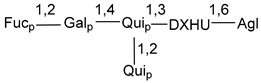	_«-----------»_	[77]
**141**	(24*S*)-Methyl- pectinioside A	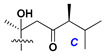	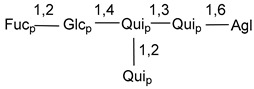	_«-----------»_	[77]
Order Brisingida Family Brisingidae *Novodinia antillensis*					
**142**	Sch 725737	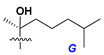	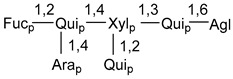	Isle de Ronde, Grenada, Grenadines, depth of 587 m	[78]
**143**	Sch 725739	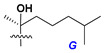	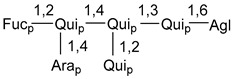	_«-----------»_	[78]

**Table 5:** Structures and taxonomic distribution of non-classical asterosaponins with one monosaccharide residue in carbohydrate chains.

№	Name	Side Chain (R)	Carbohydrate Chain (X)	Collection	References
Order Forcipulatida Family Asteriidae *Asterias forbesi*					
**147**	Forbeside E	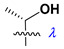	4-*O*-sulfate-Qui_p_	Indian Point, Bay of Fundy, Canada, depth was not reported	[79]
**148**	Forbeside E1	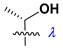	Qui_p_	_«-----------»_	[80]
**149**	Forbeside E2 (without sulfate group at C-3)	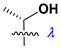	4-*O*-sulfate-Qui_p_	_«-----------»_	[80]
**150**	Forbeside E3	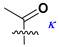	Qui_p_	_«-----------»_	[80]
*Lethasterias nanimensis chelifera*					
**151**	Cheliferoside L1	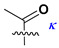	4-*O*-sulfate-Qui_p_	Isl. Shiashkotan, Kuril Islands, 100–150 m	[81]
*Aphelasterias japonica*					
**152**	Aphelasteroside C	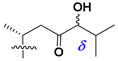	4-*O*-sulfate-Qui_p_	Posyet Bay, Sea of Japan, Russia, depth of 3–10 m	[82]
Order Paxillosida Family Astropectinidae *Astropecten latespinosus*					
**153**	Latespinoside A	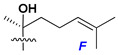	4-*O*-sulfate-Qui_p_	Hiramomijigai, Japan, depth was not reported	[83]
**154**	Latespinoside B	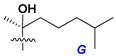	4-*O*-sulfate-Qui_p_	_«-----------»_	[83]
**155**	Latespinoside C	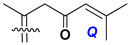	4-*O*-sulfate-Qui_p_	_«-----------»_	[83]
**156**	Latespinoside D	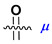	4-*O*-sulfate-Qui_p_	_«-----------»_	[83]

## References

[1] Hashimoto Y., Yasumoto T. (1960). Confirmation of saponins as a toxic principle of starfish. Bull. Jpn. Soc. Sci. Fish..

[2] Minale L., Pizza C., Riccio R., Zollo F. (1982). Steroid glycosides from starfishes. Pure Appl. Chem..

[3] Minale L., Riccio R., Zollo F. (1993). Steroidal oligoglycosides and polyhydroxysteroids from Echinoderms. Chem. Org. Nat..

[4] Iorizzi M., DeMarino S., Zollo F. (2001). Steroidal oligoglycosides from the Asteroidea. Curr. Org. Chem..

[5] Stonik V.A. (2001). Marine polar steroids. Russ. Chem. Rev..

[6] Stonik V.A., Ivanchina N.V., Kicha A.A. (2008). New polar steroids from starfish. Nat. Prod. Commun..

[7] Francis G., Kerem Z., Makkar H.P.S., Becker K. (2002). The biological action of saponins in animal systems: A review. Br. J. Nutr..

[8] Ivanchina N.V., Kicha A.A., Stonik V.A. (2011). Steroid glycosides from marine organisms. Steroids.

[9] Dong G., Xu T.H., Yang B., Lin X.P., Zhou X.F., Yang X.W., Liu Y.H. (2011). Chemical constituents and bioactivities of starfish. Chem. Biodivers..

[10] Gomes A.R., Freitas A.C., Rocha-Santos T.A.P., Duarte A.C. (2014). Bioactive compounds derived from echinoderms. RCS Adv..

[11] Ivanchina N.V., Kicha A.A., Malyarenko T.V., Stonik V.A., Gomes A.R., Rocha-Santos T., Duarte A. (2017). Advances in Natural Products Discovery.

[12] Xia J.M., Miao Z., Xie C.L., Zhang J.W., Yang X.W. (2020). Chemical constituents and bioactivities of starfishes: An update. Chem. Biodivers..

[13] http://www.marinespecies.org/Asteroidea/.

[14] Mah C.L., Blake D.B. (2012). Global diversity and phylogeny of the Asteroidea (Echinodermata). PLoS ONE.

[15] Burnell D.J., ApSimon J.W., Scheuer P.J. (1983). Echinoderm saponins. Marine Natural Products. Chemical and Biological Perspectives.

[16] Kitagawa I., Kobayashi M. (1978). Saponin and sapogenol. XXVI. Steroidal saponins from the starfish *Acanthaster planci* L. (Crown of the thorns). (2). Structure of the major saponin thornasteroside A. Chem. Pharm. Bull..

[17] Komori T., Nanri H., Itakura Y., Sakamoto K., Taguchi S., Higuchi R., Kawasaki T., Higuchi T. (1983). Biologically active glycosides from Asteroidea, 111. Steroid oligoglycosides from the starfish *Acanthaster planci* L., 2. Structures of two newly characterized genuine sapogenins and an oligoglycoside sulfate. Liebigs Ann. Chem..

[18] Itakura Y., Komori T. (1986). Biologically active glycosides from Asteroidea. X. Steroid oligoglycosides from the starfish *Acanthaster planci*. 3. Structures of four new oligoglycoside sulfates. Liebigs Ann. Chem..

[19] Komori T., Matsuo J., Itakura Y., Sakamoto K., Ito Y., Taguchi S., Kawasaki T. (1983). Biologically active glycosides from Asteroidea, II. Steroid oligoglycosides from the starfish *Acanthaster planci* L., 1. Isolation and structure of the oligoglycoside sulfates. Liebigs Ann. Chem..

[20] Ha D.T., Kicha A.A., Kalinovsky A.I., Malyarenko T.V., Popov R.S., Malyarenko O.S., Ermakova S.P., Thuy T.T.T., Long P.Q., Ivanchina N.V. (2019). Asterosaponins from the tropical starfish *Acanthaster planci* and their cytotoxic and anticancer activities in vitro. Nat. Prod. Res..

[21] Kicha A.A., Ivanchina N.V., Huong T.T.T., Kalinovsky A.I., Dmitrenok P.S., Fedorov S.N., Dyshlovoy S.A., Long P.Q., Stonik V.A. (2010). Two new asterosaponins, archasterosides A and B, from the Vietnamese starfish *Archaster typicus* and their anticancer properties. Bioorg. Med. Chem. Lett..

[22] Kicha A.A., Ivanchina N.V., Huong T.T.T., Kalinovsky A.I., Dmitrenok P.S., Long P.Q. (2010). Minor asterosaponin archasteroside C from the starfish *Archaster typicus*. Russ. Chem. Bull..

[23] Noguchi Y., Higuchi R., Marubayashi N., Komori T. (1987). Biologically active glycosides from Asteroidea, XII. Steroid oligoglycosides from the starfish *Asterina pectinifera* Müller and Troschel, 1. Structures of two new sapogenols and two new oligoglycoside sulfates: Pectiniosides A and B. Liebigs Ann. Chem..

[24] Dubois M.A., Noguchi Y., Higuchi R., Komori T. (1988). Biologically active glycosides from Asteroidea, XIV. Steroid oligoglycosides from the starfish *Asterina pectinifera* Müller and Troschel, 2. Structures of two new oligoglycoside sulfates: Pectiniosides C and D. Liebigs Ann. Chem..

[25] Dubois M.A., Higuchi R., Komori T., Sasaki T. (1988). Biologically active glycosides from Asteroidea, XVI. Steroid oligoglycosides from the starfish *Asterina pectinifera* Müller et Troschel, 3. Structures of two new oligoglycoside sulfates, pectiniosides E and F, and biological activities of the six new pectiniosides. Liebigs Ann. Chem..

[26] Iorizzi M., Minale L., Riccio R. (1990). Starfish saponins. Part 39. Steroidal oligoglycoside sulfates and polyhydroxysteroids from the starfish *Asterina pectinifera*. Gazz. Chim. Ital..

[27] D’Auria M.V., Iorizzi M., Minale L., Riccio R. (1990). Starfish saponins, part 40. Structures of two new ‘asterosaponins’ from the starfish *Patiria miniata*: Patirioside A and patirioside B. J. Chem. Soc. Perkin Trans. I.

[28] Malyarenko T.V., Kicha A.A., Ivanchina N.V., Kalinovsky A.I., Dmitrenok P.S., Ermakova S.P., Minh C.V. (2012). Asteropsiside and other asterosaponins from the starfish *Asteropsis carinifera*. Russ. Chem. Bull..

[29] Kicha A.A., Kalinovsky A.I., Ivanchina N.V., Malyarenko T.V., Dmitrenok P.S., Ermakova S.P., Stonik V.A. (2011). Four new asterosaponins, hippasteriosides A–D, from the Far Eastern starfish *Hippasteria kurilensis*. Chem. Biodivers..

[30] Riccio R., Squillace Greco O., Minale L., Pusset J., Menou J.L. (1985). Starfish saponins, Part 18. Steroidal glycoside sulfates from the starfish *Linckia laevigata*. J. Nat. Prod..

[31] Riccio R., Pizza C., Squillace Greco O., Minale L. (1985). Starfish saponins, part 17. Steroidal glycoside sulfates from the starfish *Ophidiaster ophidianus* (Lamark) and *Hacellia attenuate* (Gray). J. Chem. Soc. Perkin Trans. I.

[32] Riccio R., Iorizzi M., Squillace Greco O., Minale L., Debray M., Menou J.L. (1985). Starfish saponins, part 22. Asterosaponins from the starfish Halityle regularis: A novel 22,23-epoxysteroidal sulfate. J. Nat. Prod..

[33] Tang H.-F., Yi Y.-H., Li L., Sun P., Zhang S.-Q., Zhao Y.-P. (2005). Bioactive asterosaponins from the starfish *Culcita novaeguineae*. J. Nat. Prod..

[34] Tang H.-F., Yi Y.-H., Li L., Sun P., Zhang S.-Q., Zhao Y.-P. (2005). Three new asterosaponins from the starfish *Culcita novaeguineae* and their bioactivities. Planta Med..

[35] Tang H.-F., Yi Y.-H., Li L., Sun P., Zhang S.-Q., Zhao Y.-P. (2006). Asterosaponins from the starfish *Culcita novaeguineae* and their bioactivities. Phitoterapia.

[36] Tang H.F., Yi Y.H., Li L., Sun P., Zhou D.Z., Liu B.S. (2005). A new asterosaponin from the starfish *Culcita novaeguineae*. Chin. Chem. Lett..

[37] Tang H.-F., Cheng G., Wu J., Chen X.-L., Zhang S.-Y., Wen A.-D., Lin H.-W. (2009). Cytotoxic asterosaponins capable of promoting polymerization of tubulin from the starfish *Culcita novaeguineae*. J. Nat. Prod..

[38] Ma X.G., Tang H.F., Zhao C.H., Ma N., Yao M.N., Wen A.D. (2009). Two new 24-hydroxylated asterosaponins from *Culcita novaeguinea*. Chin. Chem. Lett..

[39] Iorizzi M., Bifulco G., de Ricardis F., Minale L., Riccio R., Zollo F. (1995). Starfish saponins, part 53. A reinvestigation of the polar steroids from the starfish *Oreaster reticulatus*: Isolation of sixteen steroidal oligoglycosides and six polyhydroxysteroids. J. Nat. Prod..

[40] Kicha A.A., Kalinovsky A.I., Ivanchina N.V., Malyarenko T.V., Dmitrenok P.S., Kuzmich A.S., Sokolova E.V., Stonik V.A. (2017). Furostane series asterosaponins and other unusual steroid oligoglycosides from the tropical starfish *Pentaceraster regulus*. J. Nat. Prod..

[41] Riccio R., Zollo F., Finamore E., Minale L., Laurent D., Bargibant G., Pusset J. (1985). Starfish saponins, 19. A novel steroidal glycoside sulfate from the starfishes *Protoreaster nodosus* and *Pentaceraster alveolatus*. J. Nat. Prod..

[42] Iorizzi M., Minale L., Riccio R., Yasumoto T. (1992). Starfish saponins, 48. Isolation of fifteen sterol constituents (six glycosides and nine polyhydroxysteroids) from the starfish *Solaster borealis*. J. Nat. Prod..

[43] Chludil H.D., Seldes A.M., Maier M.S. (2002). Antifungal steroidal glycosides from the Patagonian starfish *Anasterias minuta*: Structure-activity correlations. J. Nat. Prod..

[44] Popov R.S., Ivanchina N.V., Kalinovsky A.I., Kharchenko S.D., Kicha A.A., Malyarenko T.V., Ermakova S.P., Dmitrenok P.S. (2016). Aphelasteroside F, a new asterosaponin from the Far Eastern starfish *Aphelasterias japonica*. Nat. Prod. Commun..

[45] Ikegami S., Okano K., Muragaki H. (1979). Structure of glycoside B2, a steroidal saponin in the ovary of the starfish, *Asterias amurensis*. Tetrahedron Lett..

[46] Okano K., Nakamura T., Kamiya Y., Ikegami S. (1981). Structure of ovarian asterosaponin-1 in the starfish *Asterias amurensis*. Agric. Biol. Chem..

[47] Fujimoto Y., Yamada T., Ikekawa N., Nishiyama I., Matsui T., Hoshi M. (1987). Structure of acrosome reaction-inducing steroidal saponins from the egg jelly of the starfish *Asterias amurensis*. Chem. Pharm. Bull..

[48] Okano K., Ohkawa N., Ikegami S. (1985). Structure of ovarian asterosaponin-4, an inhibitor of spontaneous oocyte maturation from the starfish *Asterias amurensis*. Agric. Biol. Chem..

[49] Riccio R., Iorizzi M., Minale L., Oshima Y., Yasumoto T. (1988). Starfish saponins. Part 34. Novel steroidal glycoside sulfates from the starfish *Asterias amurensis*. J. Chem. Soc. Perkin Trans. I.

[50] Hwang I.H., Kim D.W., Kim S.J., Min B.S., Lee S.H., Son J.K., Kim C.H., Chang H.W., Na M.K. (2011). Asterosaponins isolated from the starfish *Asterias amurensis*. Chem. Pharm. Bull..

[51] Itakura Y., Komori T., Kawasaki T. (1983). Biologically active glycosides from Asteroidea, V. Steroid oligoglycosides from the starfish *Asterias amurensis* [cf.] *versicolor* Sladen, 1. Structural elucidation of a new oligoglycoside sulfate. Liebigs Ann. Chem..

[52] Itakura Y., Komori T. (1986). Biologically active glycosides from Asteroidea, 9. Steroid oligoglycosides from the starfish *Asterias amurensis* [cf.] *versicolor* Sladen, 2. Structure elucidation of two new oligoglycoside sulfates, versicoside B and versicoside C. Liebigs Ann. Chem..

[53] Findlay J.A., He Z.-Q., Sauriol F. (1991). Forbeside D (C_62_H_101_O_31_SNa), a new saponin from *Asterias forbesi*. Complete structure by nuclear magnetic resonance (300 MHz) methods. Can. J. Chem..

[54] Findlay J.A., He Z.-Q., Blackwell B. (1990). Minor saponins from the starfish *Asterias forbesi*. Can. J. Chem..

[55] Sandvoss M., Pham L.H., Levsen K., Preiss A., Mügge C., Wünsch G. (2000). Isolation and structural elucidation of steroid oligoglycosides from the starfish *Asterias rubens* by means of direct online LC-NMR-MS hyphenation and one and two-dimensional NMR investigations. Eur. J. Org. Chem..

[56] Sandvoss M., Weltring A., Preiss A., Levsen K., Mugge C., Wünsch G. (2001). Combination of matrix solid-phase dispersion extraction and direct on-line liquid chromatography-nuclear magnetic resonance spectroscopy-tandem mass spectrometry as a new efficient approach for the rapid screening of natural products: Application to the total asterosaponin fraction of the starfish *Asterias Rubens*. J. Chromatogr. A.

[57] Sandvoss M., Preiss A., Levsen K., Weisemann R., Spraul M. (2003). Two new asterosaponins from the starfish *Asterias rubens*: Application of a cryogenic NMR probe head. Magn. Res. Chem..

[58] De Marino S., Iorizzi M., Palagiano E., Zollo F., Roussakis C. (1998). Starfish saponins. 55. Isolation, structure elucidation, and biological activity of the steroid oligoglycosides from the Antarctic starfish of the family Asteriidae. J. Nat. Prod..

[59] Riccio R., Iorizzi M., Minale L. (1986). Starfish saponins. XXX. Isolation of sixteen steroidal glycosides and three polyhydroxysteroids from the Mediterranean starfish *Coscinasterias tenuispina*. Bull. Soc. Chim. Belg..

[60] Ivanchina N.V., Malyarenko T.V., Kicha A.A., Kalinovsky A.I., Dmitrenok P.S., Ermakova S.P. (2011). Structures and cytotoxic activities of two new asterosaponins from the Antarctic starfish *Diplasterias brucei*. Russ. J. Bioorg. Chem..

[61] Iorizzi M., Minale L., Riccio R., Yasumoto T. (1993). Starfish saponins, part 51. Steroidal oligoglycosides from the starfish *Distolasterias nipon*. J. Nat. Prod..

[62] Malyarenko T.V., Kicha A.A., Ivanchina N.V., Kalinovsky A.I., Popov R.S., Vishchuk O.S., Stonik V.A. (2014). Asterosaponins from the Far Eastern starfish *Leptasterias ochotensis* and their anticancer activity. Steroids.

[63] Levina E.V., Kalinovsky A.I., Dmitrenok P.S., Martyyas E.A., Stonik V.A. (2010). Two new steroidal saponins, hylodoside A and novaeguinoside Y, from the starfish *Leptasterias hylodes reticulata* and *Culcita novaeguinea* (juvenile). Nat. Prod. Commun..

[64] Ivanchina N.V., Kalinovsky A.I., Kicha A.A., Malyarenko T.V., Dmitrenok P.S., Ermakova S.P., Stonik V.A. (2012). Two new asterosaponins from the Far Eastern starfish *Lethasterias fusca*. Nat. Prod. Commun..

[65] Bruno I., Minale L., Pizza C., Zollo F. (1984). Starfish saponins. Part 14. Structures of the steroidal glycoside sulfates from the starfish *Marthasterias glacialis*. J. Chem. Soc. Perkin Trans. I.

[66] Diaz de Vivar M.E., Maier M.S., Seldes A.M. (2000). Labidiasteroside A, a novel saponin from the Antarctic starfish *Labidiaster annulatus*. Molecules.

[67] Roccatagliata A.J., Maier M.S., Seldes A.M., Iorizzi M., Minale L. (1994). Starfish saponins. Part 2. Steroidal oligoglycosides from the starfish *Cosmasterias lurida*. J. Nat. Prod..

[68] Vazquez M.J., Quinoa E., Riguera R., San Martin A., Darias J. (1992). Santiagoside, the first asterosaponin from an Antarctic starfish (*Neosmilaster georgianus*). Tetrahedron.

[69] Finamore E., Minale L., Riccio R., Rinaldo G., Zollo F. (1991). Novel marine polyhydroxylated steroids from the starfish *Myxoderma platyacanthum*. J. Org. Chem..

[70] Thao N.P., Cuong N.X., Luyen B.T., Thanh N.V., Nhiem N.X., Koh Y.S., Ly B.M., Nam N.H., Kiem P.V., Minh C.V. (2013). Anti-inflammatory asterosaponins from the starfish *Astropecten monacanthus*. J. Nat. Prod..

[71] De Marino S., Borbone N., Iorizzi M., Esposito G., McClintock J.B., Zollo F. (2003). Bioactive asterosaponins from the starfish Luidia quinaria and Psilaster cassiope. Isolation and structure characterization by two-dimensional NMR spectroscopy. J. Nat. Prod..

[72] Komori T., Krebs H.C., Itakura Y., Higuchi R., Sakamoto K., Taguchi S., Kawasaki T. (1983). Biologically-active glycosides from Asteroidea. 6. Steroid oligoglycosides from the starfish *Luidia maculata* Troschel. 1. Structures of a new aglycone sulfate and 2 new oligoglycoside sulfates. Liebigs Ann. Chem..

[73] Minale L., Riccio R., Squillace Greco O., Pusset J., Menou J.L. (1985). Starfish saponins-XVI. Composition of the steroidal glycoside sulfates from the starfish *Luidia maculata*. Comp. Biochem. Physiol..

[74] Krebs H.C., Komori T., Kawasaki T. (1984). Biologisch aktive Glycoside aus Asteroidea, VII. Steroid-Oligoglycoside aus dem Seestern *Luidia maculata* Müller et Troschel, 2 Die Strukturen von zwei neuen Oligoglycosidsulfaten. Liebigs Ann. Chem..

[75] De Marino S., Iorizzi M., Zollo F., Amsler C.D., Greer S.P., McClintock J.B. (2000). Starfish saponins, LVI. Three new asterosaponins from the starfish *Goniopecten demonstrans*. Eur. J. Org. Chem..

[76] Iorizzi M., de Ricardis F., Minale L., Riccio R. (1993). Starfish saponins, 52. Chemical constituents from the starfish *Echinaster brasiliensis*. J. Nat. Prod..

[77] De Marino S., Minale L., Zollo F., Iorizzi M., Le Bert V., Roussakis C. (1996). Starfish saponins. 54. Cytotoxic asterosaponins from an Antarctic starfish of the family Echinasteridae. Gazz. Chim. Ital..

[78] Yang S.W., Chan T.M., Buevich A., Priestley T., Crona J., Reed J., Wright A.E., Patel M., Gullo V., Chen G. (2007). Novel steroidal saponins, Sch 725737 and Sch 725739, from a marine starfish, *Novodinia Antill*. Bioorg. Med. Chem. Lett..

[79] Findlay J.A., He Z.-Q., Jaseja M. (1989). Forbeside E: A novel sulfated sterol glycoside from *Asterias forbesi*. Can. J. Chem..

[80] Findlay J.A., He Z.-Q. (1990). Novel sulfated sterol glycosides from *Asterias forbesi*. J. Nat. Prod..

[81] Kicha A.A., Kalinovsky A.I., Stonik V.A. (1991). Steroid sulfates from the starfish *Lethasterias nanimensis chelifera*. Chem. Nat. Compd..

[82] Ivanchina N.V., Kicha A.A., Kalinovsky A.I., Dmitrenok P.S., Stonik V.A., Riguera R., Jiménez C. (2000). Hemolytic polar steroidal constituents of the starfish *Aphelasterias japonica*. J. Nat. Prod..

[83] Higuchi R., Fujita M., Matsumoto S., Yamada K., Miyamoto T., Sasaki T. (1996). Biologically active glycosides from Asteroidea. 35. Isolation and structure of four new steroid glycoside disulfates from the starfish *Asteropecten latespinosus*. Leibigs Ann. Chem..

[84] Palagiano E., Zollo F., Minale L., Gomez Paloma L., Iorizzi M., Bryan P., McClintock J., Hopkins T., Riou D., Roussakis C. (1995). Downeyosides A and B, two new sulfated steroid glucuronides from the starfish *Henricia downeyae*. Tetrahedron.

[85] Palagiano E., Zollo F., Minale L., Iorizzi M., Bryan P., McClintock J., Hopkins T. (1996). Isolation of 20 glycosides from the starfish *Henricia downeyae*, collected in the Gulf of Mexico. J. Nat. Prod..

[86] Skropeta D. (2008). Deep-sea natural products. Nat. Prod. Rep..

[87] Skropeta D., Wei L. (2014). Recent advances in deep-sea natural products. Nat. Prod. Rep..

[88] De Riccardis F., Iorizzi M., Minale L., Riccio R., Debitus C. (1992). The first occurrence of polyhydroxylated steroids with phosphate conjugation from the starfish *Tremaster novaecaledoniae*. Tetrahedron Lett..

[89] De Riccardis F., Minale L., Riccio R., Giovannitti B., Iorizzi M., Debitus C. (1993). Phosphated and sulfated marine polyhydroxylated steroids from the starfish *Tremaster novaecaledoniae*. Gazz. Chim. Ital..

[90] Mackie A.M., Singh H.T., Owen J.M. (1977). Studies on the distribution, biosynthesis and function of steroidal saponins in echinoderms. Comp. Biochem. Physiol..

[91] Barnett D., Dean P.W., Hart R.J., Lucas J.S., Salathe R., Howden M.E.H. (1988). Determination of contents of steroidal saponins in starfish tissues and study of their biosynthesis. Comp. Biochem. Physiol..

[92] Smith A.G., Goad L.J. (1971). The metabolism of cholesterol by the echinoderms *Asterias rubens* and *Solaster papposus*. FEBS Lett..

[93] Voogt P.A., van Rheenen J.W.A. (1976). On the origin of the sterols in the sea star *Asterias Rubens*. Comp. Biochem. Physiol..

[94] Goad L.J., Scheuer P.J. (1978). The sterols of marine invertebrates: Composition, biosynthesis and metabolites. Marine Natural Products. Chemical and Biological Perspectives.

[95] Stonik V.A., Elyakov G.B., Scheuer P.J. (1988). Secondary metabolites from echinoderms as chemotaxonomic markers. Bioorganic Marine Chemistry.

[96] Den Besten P.J. (1998). Cytochrome P450 monooxygenase system in echinoderms. Comp. Biochem. Physiol..

[97] Ivanchina N.V., Kicha A.A., Malyarenko T.V., Kalinovsky A.I., Dmitrenok P.S., Stonik V.A. (2013). Biosynthesis of polar steroids from the Far Eastern starfish *Patiria* (=*Asterina*) *pectinifera*. Cholesterol and cholesterol sulfate are converted into polyhydroxylated sterols and monoglycoside asterosaponin P1 in feeding experiments. Steroids.

[98] Fusetani N., Kato Y., Hashimoto K., Komori T., Itakura Y., Kawasaki T. (1984). Biological activities of asterosaponins with special reference to structure-activity relationships. J. Nat. Prod..

[99] Yasumoto T., Watanabe T., Hashimoto Y. (1964). Physiological activities of starfish saponin. Bull. Jpn. Soc. Sci. Fish..

[100] Lucas J.S., Hart R.J., Howden M.E., Salathe R. (1979). Saponins in eggs and larvae *of Acanthaster planci* (L.) (Asteroidea) as chemical defences against planktivorous fish. J. Exp. Mar. Biol. Evol..

[101] Komori T. (1997). Toxins from the starfish *Acanthaster planci* and *Asterina pectinifera*. Toxicon.

[102] Demeyer M., Wisztorski M., Decroo C., De Winter J., Caulier G., Hennebert E., Eeckhaut I., Fournier I., Flammang P., Gerbaux P. (2015). Inter- and intra-organ spatial distributions of sea star saponins by MALDI imaging. Anal. Bioanal. Chem..

[103] Mackie A.M., Lasker R., Grant P.T. (1968). Avoidance reactions of a mollusc *Buccinum undatum* to saponin-like surface-active substances in extracts of the starfish *Asterias rubens* and *Marthasterias glacialis*. Comp. Biochem. Physiol..

[104] Yasumoto T., Tanaka M., Hashimoto Y. (1966). Distribution of saponins in echinoderms. Bull. Jpn. Soc. Sci. Fish..

[105] Ikegami S., Kamija Y., Tamura S. (1972). Isolation and characterization of spawning inhibitors in ovary of the starfish *Asterias amurensis*. Agric. Biol. Chem..

[106] Voogt P.A., Huiskamp R. (1979). Sex-dependence and seasonal variation of saponins in the gonads of the starfish *Asterias rubens*: Their relation to reproduction. Comp. Biochem. Physiol..

[107] Hoshi M., Nishigaki T., Ushiyama A., Okinaga T., Chiba K., Matsumoto M. (1994). Egg-jelly signal molecules for triggering the acrosome reaction in starfish spermatozoa. Int. J. Dev. Biol..

[108] Kicha A.A., Ivanchina N.V., Gorshkova I.A., Ponomarenko L.P., Likhatskaya G.N., Stonik V.A. (2001). The distribution of free sterols, polyhydroxysteroids and steroid glycosides in various body components of the starfish *Patiria (=Asterina) pectinifera*. Comp. Biochem. Physiol..

[109] Kicha A.A., Ivanchina N.V., Stonik V.A. (2003). Seasonal variations in the levels of polyhydroxysteroids and related glycosides in the digestive tissues of the starfish *Patiria (=Asterina) pectinifera*. Comp. Biochem. Physiol..

[110] Popov R.S., Ivanchina N.V., Kicha A.A., Malyarenko T.V., Grebnev B.B., Stonik V.A., Dmitrenok P.S. (2019). The distribution of asterosaponins, polyhydroxysteroids and related glycosides in different body components of the Far Eastern starfish *Lethasterias fusca*. Mar. Drugs.

[111] Lazzara V., Arizza V., Luparello C., Mauro M., Vazzana M. (2019). Bright spots in the darkness of cancer: A review of starfishes-derived compounds and their anti-tumor action. Mar. Drugs.

[112] Katanaev V.L., Di Falco S., Khotimchenko Y. (2019). The anticancer drug discovery potential of marine invertebrates from Russian Pacific. Mar. Drugs.

[113] Verbist J.F., Jangoux M., Lawrence J.M. (1993). Pharmacological effects of compounds from echinoderms. Echinoderm Studies.

